# Contagious Pleuro-pneumonia in Goats at Cape Colony, South Africa

**Published:** 1890-03

**Authors:** D. Hutcheon

**Affiliations:** Colonial Veterinary Surgeon, Cape of Good Hope


### CONTAGIOUS PLEURO-PNEUMONIA IN GOATS AT CAPE COLONY, SOUTH AFRICA.

By D. Hutcheon, M.R.C.V.S., Colonial Veterinary Surgeon, Cape of Good Hope.

[*Veterinary Journal, London, Dec., 1889.*]

In a report on a fatal disease among goats in the district of Khandeish, by Principal John Henry Steel, which appeared in the *Veterinary Journal* (No. 171, p. 153), he refers to a report of mine on "Contagious Pleuro-pneumonia in Goats in Cape Colony," which appeared in the same Journal a few years ago (No. 13, 1881, p. 171). As that report was written at an early period of the outbreak of the disease referred to, it was, in consequence, very incomplete. I beg leave, therefore, to supply a little additional information respecting it, taken principally from my report to the Cape Government after the disease had been entirely suppressed in the colony.

I will not occupy space by giving a detailed account of all the experiments conducted during the progress of that virulent scourge, but I will simply state the facts which these experiments elicited; and as the disaase has ceased to exist in South Africa, I will refer to it in the past tense.

(1) *Respecting the Nature of the Disease.*—It was a specific infectious form of pleuro-pneumonia, affecting goats only, cattle and sheep remaining free from infection, although constantly exposed to it. The disease was introduced into the Cape Colony by a shipment of Angora goats from Asia Minor, where the disease is represented as being indigenous.

(2) *The Manner in which the Disease was Spread.*—The disease was carried from flock to flock by the near contact of affected and healthy goats, a very limited space being sufficient to prevent it spreading from the one to the other. In fact, I had no proof that the disease could be communicated in any other manner than by the immediate contact of the diseased with the healthy. Neither kraals nor camps appeared to retain the infection after the disease had ceased in the flocks frequenting them.

Healthy goats placed amongst an infected flock one month after the last case of disease occurred in it, became affected with the malady. These were immediately destroyed, and the same test applied in another flock two months after the disease had ceased. In this experiment the healthy goats remained free from any symptoms of the disorder. In fact, with one exception, in which the history of the flock could not be correctly ascertained, there was no re-appearance of the disease due to the introduction of healthy goats into a previously infected flock, although numbers were so introduced after it had ceased from three to six months only.

(3) *Respecting Inoculation as a Preventive.*—The following statement will indicate its value :—

| | |
|---|---|
| The total number of goats inoculated was . . . . . . . | 44,000 |
| The total number inoculated a second time . . . . . . | 20,000 |
| The total number inoculated a third time . . . . . . . | 1,600 |
| Total number of inoculations . . . . . . . . . . . . | 65,000 |

The number of flocks which were affected with the disease, and in which no inoculations took place, was 19, containing a total of 7,500 goats ; of these 5,100 died of the disease.

The number of flocks which were inoculated *after* the disease had appeared amongst them was 12, containing a total of 12,550 goats. Of these 4,380 died.

The number of flocks which were inoculated previous to any disease appearing amongst them, but in which it subsequently manifested itself, was 35, containing a total of 21,500 goats. Of these 2,860 died of the disease.

The number of flocks which were inoculated, which were free from disease at the time of inoculation, and in which no disease followed, was 18, containing a total of 9,950 goats.

From the foregoing statement, it will be apparent that when the disease was allowed to run its course in a flock, unchecked by any preventive measures, about two-thirds died. When inoculation was resorted to as soon as the disease was observed in a flock, less than one-third died. The average number, it will be observed, is higher, but that is due to the fact that very few flocks were inoculated on the first appearance of the disease amongst them ; a considerable number were generally affected before inoculation was resorted to, especially before the beneficial effects of inoculation became generally known.

Of six healthy goats inoculated and placed immediately after the operation in a flock of goats in which the disease was raging, five took the disease and died, and only one escaped the infection. Of seven healthy goats inoculated, and kept for twelve days before being placed in a flock of diseased goats, one only took the disease, the remaining six showed no symptoms of it.

It will be observed that a comparatively large number of goats died, even in those flocks which were inoculated previous to the appearance of any disease amongst them. The mortality under this heading occurred principally in ewe flocks. In ewes considerably advanced in pregnancy, inoculations communicated the disease to the fœtus, and abortion followed. Many of these kids were born alive ; they would linger for a few days, and die of the disease. The inoculation was thus the means of introducing the disease to thirty-five flocks, principally through the medium of the diseased kids. This led to the discovery of another very important fact, viz., that the preventive effects of inoculation only lasted from four to six months. As soon as this fact dawned upon me, I recommended the re-inoculation of all flocks in which the disease re-appeared, and the prompt slaughter of all that became affected. This plan proved a complete success, except in one instance, which I will here relate. The disease made its appearance at a farm called Groonkloof, in the Waterkloof district of the Fort Beaufort Division, on September 20th, 1881. There were 1,460 goats on the farm, belonging to three different owners. Mr. Hartzenberg owned 1,000 ; Mr. du Preez, 260 ; and Mr. Heath, 200. The disease appeared in Mr. Heath's goats. I inoculated the three flocks on September 23d. The disease passed through Mr Heath's flock, carrying off about the half of them, but no disease manifested itself in Messrs. Hartzenberg or Du Preez's goats until some time after, when individual cases began to occur. I then re-inoculated these two flocks, using a large dose of virus, and was considerably disappointed to find that even then the disease did not subside. I then inoculated them a third time, again using a large dose of virus, when, to my dismay, on examining the flock a fortnight after, I found that nearly every goat was coughing and exhibiting slight symptoms of the disease. About one hundred took the malady in a severe form. These were picked out and destroyed. The disease, however, kept lingering in this flock, although very few died of it, until July, 1882, when twelve cases occurred in about as many days. The disease had been practically stamped out in every other flock in the Colony several months prior to this. Authority was, therefore, obtained, and the whole flock was killed, and their carcases buried on August 8th. The scourge has not appeared since.

In my opinion, I communicated the disease to this flock direct, by using too large a quantity of virus in the inoculations. This opinion was confirmed by direct experiment, for I found that I could communicate the disease to a large percentage—3 in 12 inoculated—by injecting a large quantity—12 minims—of the virus. The disease was also communicated to a large number by taking a small wineglassful of the fluid found in the chest of an infected goat and administering it as a drink to a healthy one. Again, I inoculated two other small flocks of goats three times. In the first two inoculations, I used about 5 minims of virus to each inoculation, and about 8 minims the third time I inoculated them. Two kids only became affected in these flocks ; these were killed, and no further indications of the disease appeared.

Many mistakes were made during the course of the plague, but which could hardly be avoided, as I was working tentatively for a considerable time. The following are the directions which I gave in my report upon the subject.

In preparing the virus, select a goat which appears to be suffering from the disease in the acute stage, just when it commences to give the peculiar grunt at each expiration. After cutting its throat and allowing it to bleed, open the chest by running a knife through the cartilages of the ribs on each side of the sternum, taking care not to cut through the bloodvessels entering the cavity. Lift off the sternum, then turn over the goat and pour out the fluid contained in the thorax into a dish. After doing this, cut out the diseased lung or lungs. If the diseased lung is suitable for inoculation, it will present a bluish slate-color, and be in juicy condition when cut through with a knife. Any portions that have a dirty, yellowish-grey appearance must be rejected. But when degenerative changes have taken place in the diseased lung, it becomes dry; so that little virus can be obtained from it.

There is no thickening of the interlobular tissue in the diseased lung of the goat, and which forms such a striking feature in bovine pleuro-pneumonia. The section of a diseased lung in the goat has the appearance of a somewhat granular-looking liver.

In expressing the virus from the lung, I cut it into thin slices, and expressed the juice from these through a piece of open sacking. I then restrained both the fluid expressed from the lung and also that obtained from the thorax through a piece of muslin, mixing them together.

I used a small hypodermic syringe, with strong needle points, for inoculating, injecting the virus under the skin of the inner surface of the tail.

If the goats are free from disease at the time of inoculation use 5 minims of the virus for a first inoculation. If they are still exposed to the infection, re-inoculate them in from one to two months' time, using 8 minims of the virus for the second inoculation. Should the disease be present among the goats previous to inoculating, it is better then to use about 8 minims of the virus, as a second inoculation is not required.

*History of the Disease in Cape Colony.*—The disease appeared amongst Mr. Van Vickerks' goats, in the Bedford district, early in March, 1881. Three days after, when I arrived at the conclusion that the disease was both special and contagious, I recommended the immediate destruction of the affected flock, but the Government did not see its way to carry out such a policy. Very soon the disease extended to a large number of flocks, after which the farmers did not think it possible to eradicate it. Seeing that there was little hope of getting the Government to carry through a stamping-out policy, I set energetically to work to try to arrest its spread by means of inoculation. Meanwhile an act was passed empowering the Government to order the slaughter of infected goats; but no compensation was allowed in the case of animals affected with the disease. Compensation was only authorized when it was considered necessary to destroy healthy animals. The result was that the act was comparatively useless. No "Board" would order the slaughter of infected animals without having the power to grant compensation.

So matters remained until October, 1881, when, by persistent pegging away, I got a deputation to meet the Commissioner of Crown Lands and myself. At this meeting, I showed that the disease could be stamped out at a very moderate cost if I was allowed to adopt a certain course. He granted the request of the deputation, and authorized me to proceed in the manner I had indicated, which was as follows :—

Where the disease appeared in a flock which had been inoculated, we immediately selected all affected goats, and had them destroyed, re-inoculating the whole of the remainder. Any goats that showed signs of the disease after the second inoculation were also promptly destroyed. In every instance except the one already described, this second inoculation arrested the further spread of the disease almost immediately. Where the disease appeared in a flock which had not been previously inoculated, we had the whole flock slaughtered immediately. In this manner, 3,531 goats and 2,311 kids were destroyed, and the disease was practically arrested by the end of December of the same year.

